# Supplementary figures and images for: Ensuring the quality and specificity of preregistrations
Source: PLoS Biol. 2020 Dec 9;18(12):e3000937. doi: 10.1371/journal.pbio.3000937 (PMC7725296; doi:10.1371/journal.pbio.3000937)

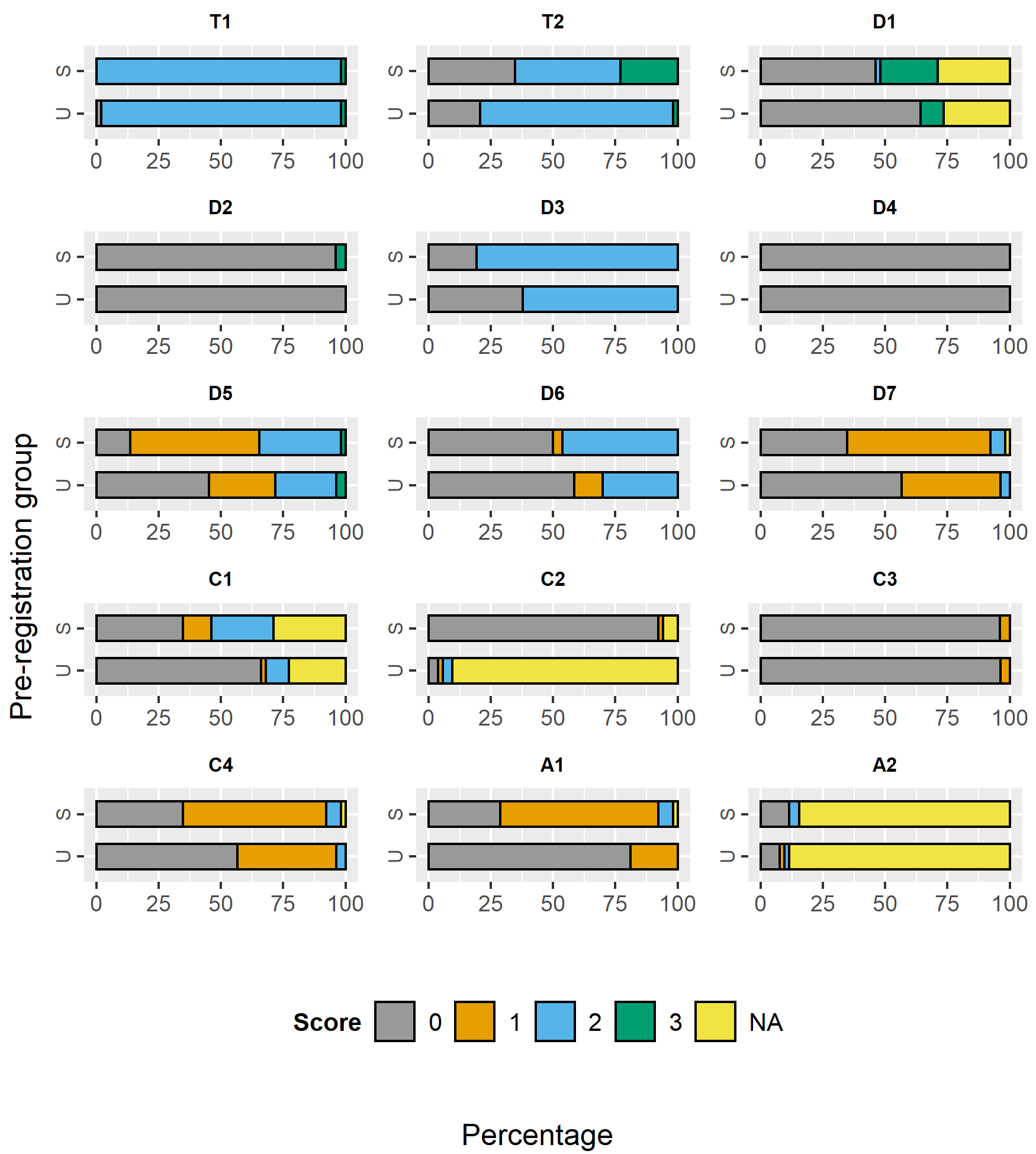

Supplement: S1 Fig — The data underlying this Figure can be found at https://osf.io/fgc9k/. (TIFF) [file pbio.3000937.s001.tiff]

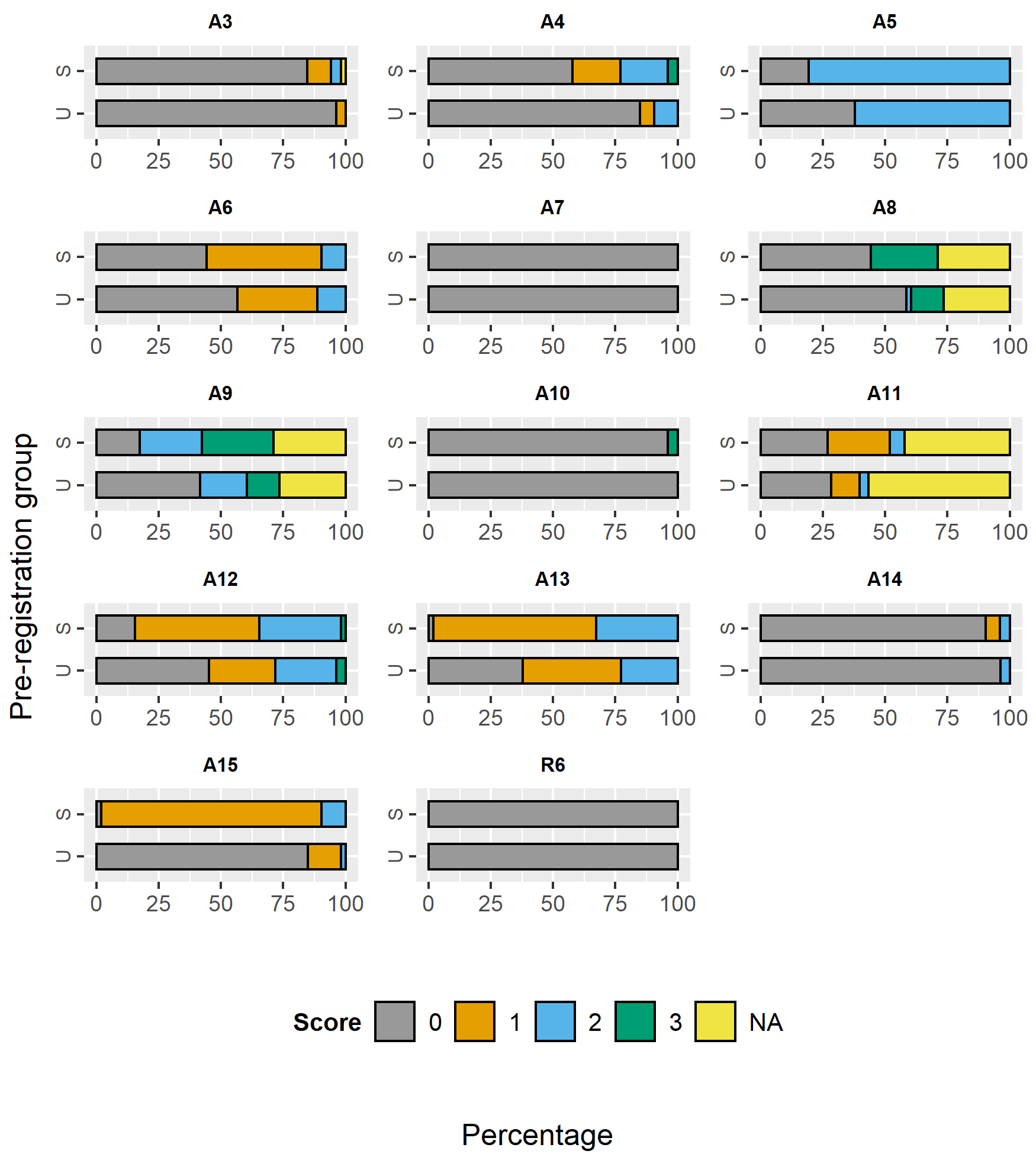

Supplement: S2 Fig — The data underlying this Figure can be found at https://osf.io/fgc9k/. (TIFF) [file pbio.3000937.s002.tiff]
