# Supplementary material for: Ensuring the quality and specificity of preregistrations
Source: PLoS Biol. 2020 Dec 9;18(12):e3000937. doi: 10.1371/journal.pbio.3000937 (PMC7725296; doi:10.1371/journal.pbio.3000937)
Supplement: Attachment — Submitted filename: 20200221_Reaction on reviews.docx [file pbio.3000937.s003.docx]

*We include the letter of the editor with the reviewers in normal text, while our response to the letter is in italics.*

Dear Dr. Veldkamp:

Thank you for submitting your Empirical Article (AMPPS-18-0115) entitled "Ensuring the quality and specificity of preregistrations" to Advances in Methods and Practices in Psychological Science (AMPPS). I have now received reviews from three researchers who are highly familiar with this topic and the broader conversation space surrounding it. I have read and evaluated your manuscript as well. I apologize for the delay in this response – as you will see, the reviewers were quite mixed in their overall assessment of the manuscript and its suitability for publication. They raise several topics that are quite nuanced and complex. In considering my own response, I read the paper multiple times both before and after reading the reviewers’ comments. Ultimately, I would like to leave the door open for submission of a major revision, but must emphasize that a revision may not ultimately be accepted for publication and I would likely enlist additional responses from at least one of the previous reviewers to evaluate a revised manuscript in its new form. I do think the topic is incredibly important and timely, but ultimately also agree with many of the reviewer concerns about the current version of the manuscript.

The reviewers offer many thoughtful and comprehensive points for consideration, which I will not reiterate here. I would like to see careful and detailed responses to each of their points in a potentially revised manuscript and accompanying cover letter. Reviewer 1 and Reviewer 2 raise a number of especially challenging concerns that point to more substantial avenues for revision. In my reading, these comments highlight questions of 1) actual utility – how do we know that studies pre-registered using the more detailed protocol are actually “better” in any variety of ways and 2) the potential for iatrogenic effects, including but not limited to initial deterrence. Ideally, actual data should be brought to bear on these two points in a revised manuscript. At a minimum, they should be very clearly discussed and operationalized in the paper – I would want to know by what exact criteria the authors anticipate these two points could be measured, although I am not sure even that sort of discussion would be sufficient to address them. I leave it to the authors’ consideration how to most effectively tackle some of these more challenging issues, should they choose to pursue a revision. I think these deeper issues are also connected to the reviewer consensus that the finding ultimately seemed quite obvious (“more detailed pre-registrations were associated with more constrained researcher behavior”), to the extent that the reviewers indicated a clear desire for more impact/take-home from the paper. The reviewers raise a number of other comments, as well, all of which I would like considered carefully by the authors, should you choose to submit a revised manuscript.

*We agree that answering the important question ‘Does pre-registration lead to better science?’ should be a final empirical goal. However, answering such a question cannot be answered by a single research team. It will be a time-consuming research enterprise, involving many researchers or teams. We are currently preparing a study in which we willcompare articles of ‘research as usual’ (not preregistered) with articles that were preregistered with different formats, and our estimates of coding time alone vary from 1,500 to 3,500 hours. Only the coding! We also expect that the report of such findings will be quite extensive, deserving of its own paper.*

*We therefore believe it is not sensible to include, in one paper, both the quality (i.e., the extent to which they restrain a number of degrees of freedom) of preregistrations (present paper) and the quality of the final article (important, future work).*

*Our revised and improved paper is focused on the evaluation of the quality of the preregistrations of different formats.*

If you choose to submit a revision, please include include a letter detailing your point-by-point responses to each reviewer comment and indicating how you changed the manuscript to address them. The revised manuscript may undergo further peer review. We ask that you submit your revision within three months. Please let us know if you will not be able to meet this deadline.

To submit your revision, log into <http://mc.manuscriptcentral.com/ampps> and enter your Author Center, where you will find your manuscript title listed under "Manuscripts with Decisions." Under "Actions," click on "Create a Revision." Your manuscript number has been appended to denote a revision.

IMPORTANT: Your original files are available to you when you upload your revised manuscript. Please delete any redundant or outdated files before completing the submission.

Once again, thank you for submitting your manuscript to AMPPS and I look forward to receiving your revision.

Sincerely,

Jennifer Tackett
Associate Editor, Advances in Methods and Practices in Psychological Science
[jenniferltackett@gmail.com](mailto:jenniferltackett@gmail.com)


Reviewer(s)' Comments to Author:
Reviewer: 1

Comments to the Author
The current study compares two preregistration templates – the Preregistration Challenge Registration (PCR) and Standard Pre-Data Collection Registrations (SPC) – on the degree to which they restrain a number of researcher degrees of freedom. The current study is an excellent example of a thoroughly pre-registered study, and the data, methods, and results were of high quality. To be frank, it was refreshing to read a study where the authors clearly stated their intentions, documented their process and followed through on their plans. I compared the preregistered R script to the final one and found no deviations (excepting additions for calculating descriptive statistics and a few minor cleaning adjustments). However, I have some concerns regarding the justification and conclusions of this study that temper my enthusiasm.

Major concerns
1.      At the end of reading this manuscript, I had trouble articulating what question this study addressed and what I was supposed to learn from it. In the introduction, the authors describe what a preregistration is and what it should accomplish; from there, they jump right to the current study section. This was jarring, and more importantly, it was not clear to me at this point what the goal of the study was in a larger, theoretical sense. Were the authors trying to evaluate the effectiveness of preregistration templates? Were they trying to compare templates to figure out which one was best? Further confusing: this section includes hypotheses that are not justified by the literature cited – why would we expect the PCR to receive higher Restriction Scores than the SPC? It wasn’t clear to me what this study was trying to accomplish, why it had chosen to compare these two templates, or what the analyses would tell us about preregistration writ large. Answering these questions would go a long way towards improving the quality of this study.

*We agreed with the reviewer that (due to the previous word limits) the transition from the introduction to the methods section was not clear. We rewrote and expanded the introduction by discussing the different preregistration formats more extensively and added the research questions.*

2.      I am concerned that authors are overinterpreting the results of this study. More specifically, throughout the manuscript, the authors describe the restriction of researcher degrees of freedom by these templates. But what goes unaddressed is the degree to which researchers actually stick with what they preregistered. Preregistration templates can provide transparency (given that they meet the criteria of being specific, precise, and exhaustive and are also publicly available); but the reality is that restriction comes from institutions. Reviewers who compare preregistrations to submitted manuscripts; editors who refuse to publish non-preregistered studies or studies that deviate from preregistrations; funding agencies that require and monitor preregistration; societies who encourage and reward preregistration; and teachers and mentors who train preregistration are the ones who restrict our researcher degrees of freedom. The templates are merely a tool to aid in this. What have we learned from this study? I believe we have learned that the PRC template asks more and more detailed questions than the SPC template. But we have not learned whether the resulting studies are of higher quality or produce more robust results. Nor have we established that researchers who choose to use a more detailed or exhaustive template are not more motivated to produce higher quality and more transparent work. Nor have we learned that asking more and more detailed questions in preregistrations will not deter researchers from engaging in preregistration. All in all, the results of this study are intuitive (more detailed templates yield more detailed preregistrations) but they do not support the (unspecified) hypothesis that a more detailed preregistration template is more effective. This issue could be partially resolved by clarifying the definition of “effective” throughout the manuscript. This would help to address my point above (what are we trying to learn) and also circumscribe the conclusions drawn.

*We agree with the reviewer that preregistration is a tool, and that in the end, we would like to learn the extent to which the tool aids in improving science and its reproducibility. As explained in our response to the editor, we believe that assessing the extent to which preregistration ultimately improves science and its reproducibility amounts to a large research program, which is not suited for one study and paper.*

*We do not agree that we learned from our study that ‘the PRC template asks more and more detailed questions than the SPC template’. The main message from our study is that preregistrations, in general, lack quality (e.g., coders only agreed in 15% of preregistrations on their number (!) of hypotheses) and do not restrain researcher degrees of freedom well.*

*As we agree with the reviewer that the use of ‘effective’ is awkward, we deleted it from most of the text. We restrict ourselves to the extent to which current preregistrations restrict using researcher degrees of freedom.*

*In our discussion, we now also mention that the changes should be extended to other stakeholders as well. However, we also indicated that for these changes to occur, these institutions need good protocols and examples of what a good preregistration should contain.*

Minor concerns:
1.      I could not find the raw data (final_analysis_data.csv) on OSF, only the cleaned version of the data frame inside the Rdata file. Please provide the raw data too!

*We have added the raw data also to the OSF*

2.      I am concerned about the number of analyses performed in this study. Preregistration, unfortunately, does not guard against family-wise error. I understand not wanting to adjust p-values, given that that was not included in the preregistration. However, nearly half of the significant results (6 of 14) fell in the range .01 < p < .05. I would recommend reporting both original p-values and adjusted p-values. To that aim, I would also recommend using a Holm correction.

*As suggested by the reviewer we’ve added the Holm corrected p values to Table 2, but also made clear in the text that this correction was not preregistered.*

3.      Table 2 reports the means of each DF for each type of format, but the statistical test is a test of medians. Please also report the medians in this table, especially if you anticipated non-normal distributions.

*We did not add the medians, since we present the complete distribution of the scores in Table 2, by giving the percentage of preregistration that received each score (0, 1, 2, and 3). Therefore, readers can infer the median easily, and also reporting the medians will not be informative. Note that the medians of the restriction scores are given in the text.*

4.      What is the justification for using the PRC template versus another kind of template that is registered on OSF? More importantly, how should readers use the results of this study to inform their choice of template, especially in light of the fact that the PRC template will soon be revamped?

*We added to the introduction more information about the choice of the two templates. SPR is the only unstructured format that asked the researcher to indicate that the registration is done before collecting the data. PCR was the format that was most extensive and because of the preregistration challenge also often used.*

*We added to our discussion that we would like to investigate other templates as well. We do not see the problem of the updating of the PCR template. Our main result is that more extensive templates result in preregistrations that prevent the opportunistic use of researcher degrees of freedom better. However, we also showed that the PCR too should be further improved. Therefore, our findings will be used to improve the current format.*

Reviewer: 2

Comments to the Author
I consider it very important to work on ways to improve the quality and specificity of pre-registration, so I read this paper with great interest. However, my reaction was mixed, and I found myself disagreeing with some of the choices made, although it's possible that a more detailed analysis of this archival material might give clearer information.

1.      I would like to see more background about the PCR format – how it was developed, whether it was developed independently from the Wicherts et al. (2016) degrees of freedom list that is used as an assessment criterion, what elements it specifically enforces. We jump right into the analysis, and the prediction that it will be better than the standard, with very little description.

*As stated as well at point 1 of Reviewer 1, we rewrote and expanded the introduction to create a better transition between introduction and methods section and better describe the preregistration formats. The development of PCR was independent of the list of researcher degrees of freedom by Wicherts et al. (2016). The co-authors of this paper represent some convergence of the Wicherts et al. (2016) team and the designers of the PCR (and SPR) formats – BAN, CKS, and DM.*

2.      It is not clear from the Method or the preregistration whether PCR protocols were evaluated at the point when they had not yet been vetted by the Prereg Challenge staff. If not, this introduces another confound in the PCR-SPR comparison: the “intervention” would consist both of preparing to a standard and then having that standard checked rigorously.

*The PCR protocols were evaluated* ***after*** *they were vetted by the Prereg Challenge staff. We made this clear in the methods section. However, this checking was superficial; for completeness only instead of an in-depth evaluation of the substance (all included PCRs were registered and approved for the preregistration challenge after verifying that the form was filled out completely). Nevertheless, researchers might have tried harder to write a good preregistration because they knew that they were being checked. We discuss this as a limitation in our discussion, but also make clear that to make checking possible, we also need to know what a good preregistration should contain. Furthermore, also the people who did use the SPR format would know that their preregistration would become part of what is reviewed after submission of the final paper.*

3.      The list of 34 “degrees of freedom” in my opinion deviates somewhat from the definition of researcher degrees of freedom (RDF). These, for me, are decisions made in a way that can affect the outcome and conclusions of statistical tests after data are collected. But the list mixes in a number of things that, while good research practices, cannot be used to p-hack data. For example, randomizing participants to condition ad hoc, having non-blinded experimenters, or failing to report a power analysis, cannot influence the analysis of data once collected. Things like outlier exclusion, additional data collection, or moving the hypotheses to meet the data, on the other hand, are more commonly understood as RDF in this sense.

*We agree with the reviewer that we can discuss whether all of the RDFs of the list of Wicherts et al. (2016) are ‘true’ RDFs. Exploratively, we recalculated the restriction scores without three items that arguably may not be related to p-hacking (D6, failing to conduct a power analysis; C1, failing to randomly assign participants to conditions; C2, insufficient blinding) and present the results in the paper. We found comparable results (even with a slightly larger effect) after removing these RDFs.*

4.      My other concern is that the strongest results seem to come from reporting elements that are not standard in published research, but (as indicated when the results are presented) may only be reported when a protocol asks for them. For example, the randomization method, or the fact that outliers are not dealt with in any particular way, is implicit in most publications. I would only consider it a failure if an outlier method was not reported in the preregistration and then resorted to post-hoc in the article – at least, it is impossible to tell otherwise whether outlier handling was considered as a way to p-hack if it is not ultimately reported. The criterion here is too close to the procedure; people are being asked to do things, and so they are, but the extent to which this prevents post-hoc flexibility in final reporting is not looked at closely enough for my taste. Also, as stated in point 3 I would not consider randomization or other points of good experimental design (as opposed to potentially selective analysis) as being at all germane to the stated goals of pre-registration. Improving experimental design in this way is more of a side benefit. Therefore I did not get a good overall picture of whether the PCR format was effective in restricting the practices that are really important to p-hacking, as the overall figure combines many things, and the individual p-values are hard to combine meaningfully.

*We agree with the reviewer that some elements of research, like using randomization or not, or more characteristic of how to do (good) research, than necessary elements of preregistration per se. What counts is whether preregistrations restrain researcher degrees of freedom or not, and whether preregistrations are specific enough by telling what they exactly plan to do (thereby restraining RDFs).*

*As discussed in the previous point, we added an analysis of the extent to which RDFs are limited without D6, C1, and C2. We also make it more clear that if researchers do not report something in their preregistration (e.g., handling of outliers) it is unclear whether they forgot to do this, or that this implies that they will include all data. That is, it does not preclude the use of RDFs.*

*We agree that it is important and valuable to investigate further if preregistrations not mentioning something will actually do something or not in the final paper (e.g., handling of outliers), but therefore we would also need to look at the final papers. That was beyond the scope of the current paper and therefore we discuss this as a direction for further research (we are currently working on this project, see our earlier responses).*

5.      Some minor points.
a.      It’s “Giner-Sorolla” not “Sorolla” (p. 4)

*Oops, sorry! We changed this.*

b.      “Data handing” is probably meant as “data handling” (p. 9)

*We changed this.*

c.      “We excluded five items from the reporting phase of research because they could not be assessed based on the preregistration.” – I would like these items indicated in the list so the rationale can be looked at.

*We added them in the text*

d.      Fig. 1 is an odd choice given that figures should be reserved to illustrate the most important points of a manuscript. To me it gives more of the sense of promoting OSF than illustrating the main point (which itself could be clearer).

*We removed the figure from the manuscript and briefly discuss the numbers in the text.*

Signed, by policy,

Roger Giner-Sorolla

Reviewer: 3

Comments to the Author
This is a signed review (Chris Chambers, Cardiff University). Veldkamp and colleagues present an impressive observational study of the specificity, precision and exhaustiveness of preregistered protocols in a sample of self registered protocols (SPR) compared with those submitted via the COS preregistration challenge (PCR).  As expected, they found that the prereg challenge performed better at restricting potential research bias (RDFs).

This is an excellent paper. The question is timely and important; it is the first study of its kind in basic sciences to perform this kind of analysis, and with a few minor caveats (discussed below), I believe the transparent, preregistered methodology underpinning the analysis meets the authors’ challenge of ensuring rigour in preregistered study designs.

In my view the article should be published almost as-is, though I offer some recommendations for providing additional detail about the methods in the manuscript and surfacing of some additional limitations and points of discussion.

1. Identity of the coders and risk of coding bias
Where a crucial study DV arises from subjective coding or content analysis, it is generally recommended to blind coders as much as possible to the study hypotheses and group assignments (in this case whether the protocol was an SPR or a PCR). At present it isn’t clear from manuscript whether these conditions are met. I discovered from piecing together information from the manuscript and protocol that they are likely not met and there is also some ambiguity about how many coders there were in total. The manuscript states that there were two coders but various R scripts (e.g. <https://osf.io/r9x38/>) suggest that as many as five of the authors contributed to the coding. The identity of the coders should be made clear, and any deviation from the protocol also made clear (the protocol itself differs from both, suggesting three coders).

*Thank you, we agree. We’ve explained the coding procedure in more detail in the text. Indeed, there were five coders, but each preregistration was only coded by two coders. We identify the coders. Blinding was not possible as SPR and PCR use very different formats, which we also mention in our revision.*

The identity of the coders is important because of the issue of blinding. If as it seems, all coders were members of the authorship team then there is a risk of coding bias. Unless I missed it, there does not appear to have been any blinding mechanism in place to, for instance, extract the key text from each protocol, place it within a neutral template for coding that redacts information about whether it was SPR or PCR (thus blinding its source), before then being passed to the main coders. Therefore, we might ask to what extent the authors’ own expectation that PCR would outperform SPR could have influenced the results, especially given the ambiguity and lack of concordance in coding many of the protocols and RDF categories. I don’t think it is a fatal concern provided the complete data are made available for verification, so that other researchers could, if they wish, precisely duplicate the coding methodology (see next point).

*We made clear that blinding was not possible and mention this as a limitation of the current study.*

2. Data availability
All raw data should be publicly archived to ensure that the methods are reproducible. This seems especially important in light of the risk of coding bias. I may have missed this, but searching through the OSF project and URLs listed in the manuscript, I couldn’t find a list of the studies included in each sample of 53 protocols together with the analysed text of those protocols (which should also be archived in case of loss/change elsewwere) and the individual ratings supplied by each coder. I would strongly recommend that this data be archived on the OSF page.

*We added all raw data to the OSF.*

3. Correction for multiple comparisons
I was a little surprised to see no apparent consideration of multiple comparisons and alpha correction in what amounts to 29 separate hypothesis tests for each of the RDFs (preregistering the protocol helps, but of course doesn’t eliminate risk of false positives). By my count, with a Bonferroni correction applied, only 4 of the 29 RDFs would differ significantly between the SPR and PCR groups. Bonferroni correction may be too stringent (e.g. Holm-Bonferroni may be more appropriate) but in any case I would recommend some mention of this issue and perhaps identify in an exploratory analysis which DFs showed sufficient strongly group differences to survive such correction.

*Yes, reviewer 1 had a similar point (point 2). We’ve added the Holm corrected p values to Table 2. We also mention these in the text, but also made clear in the text that this correction was not preregistered.*

4. Lack of a baseline group of regular articles. Though it is not a flaw, as such, given the research question, I think the study would have been more informative if it had also included a sample of regular unregistered articles to place alongside the SPR and PCR protocols (sampled randomly within constraints dictated by the topic etc of the SPR and PCR protocols). This would have revealed to what extent preregistration per se restricts RDFs (with the caveat that HARKing etc could provide a false signal of rigour in regular articles).

*We agree that it is important and valuable to investigate this further, but for this we would also need to look at the final papers. That was outside of the scope of the current paper and therefore we discuss this as further research (we are currently working on this project, see also our previous responses).*

Other comments
I found the low concordance rate among coders about the number of hypotheses  striking and concerning, especially for PCR – and the fact that it wasn’t higher for PCR vs SPR is doubly remarkable given the more modular protocol format of PCR. This is surely a very loud wake up call for all researchers, and I would perhaps consider amplifying this particularly result in the abstract. I agree wholeheartedly with the authors that preregistering is an acquired skill and one that we need to make easier for authors learn and implement. For me (and I’m just waxing on now rather than recommending any particular revision), it strongly suggests the need for a software tool that authors can use to build a specific, precise and exhaustive protocol, rather than relying on traditional forms of a manuscript preparation. This could include a standardised template in which authors enumerate their hypotheses, linking each to a sampling plan, analysis plan and contingent interpretation, while also confirming each of the statements requiring to score 3 in the (excellent) coding scheme devised by the authors. The tool could provide warnings of incomplete/imprecise specifications and then generate a manuscript out of the responses, which could then be uploaded onto a repository or submitted as a Registered Report. Although the authors understandably didn’t include a sample of RRs in the current study, my working hypothesis (given my experience as RR editor and RR author) is that RRs “1.0” will suffer many of the same failings as identified in the PCR mode especially because Stage 1 peer review (even at its best) is too blunt an instrument to ensure specificity, precision and exhaustiveness of preregistered protocols. The authors' work here provides a strong case for the need innovate to preregistration 2.0.

*Thank you. We incorporated the low concordance rate in the abstract. We also include in our manuscript a discussion of a tool as suggested, working towards preregistration 2.0.*
