# Supplementary material for: Ensuring the quality and specificity of preregistrations
Source: PLoS Biol. 2020 Dec 9;18(12):e3000937. doi: 10.1371/journal.pbio.3000937 (PMC7725296; doi:10.1371/journal.pbio.3000937)
Supplement: Attachment — Submitted filename: Reply v4.docx [file pbio.3000937.s005.docx]

Dear dr. Wijayatilake,

We would like to thank you and the reviewers for the valuable comments on our previously submitted manuscript (PBIOLOGY-D-20-00441R1). We submitted our revised manuscript in which we dealt with all the issues raised by you and the reviewers. Please see our reply to the reviewers below (in italic).

Thanks again for considering our paper in PLOS Biology.

Best, also on behalf of the co-authors,

Marjan Bakker

Dear Dr Bakker,

Thank you very much for submitting your manuscript "Ensuring the quality and specificity of preregistrations" for consideration as a Meta-Research Article at PLOS Biology. Your manuscript has been evaluated by the PLOS Biology editors, an Academic Editor with relevant expertise, and by two PLOS Biology reviewers. Firstly, let me sincerely apologize for the unusual and lengthy delay getting you this decision. As I had explained in an earlier email, we had a reviewer signed up who, despite assurances, never delivered his/her comments. We then had to get new reviewers signed on at that late stage. We had two reviewers signed on with the aim of getting 1-2 additional reviewers, as mentioned, and luckily both have now delivered on schedule. The good news is that both are quite positive about the study and find the research question interesting. Considering these reviews, along with the reviews from Advances in Methods and Practices in Psychological Science, especially Reviewer 3 (who had signed his review), we will be happy to pursue the manuscript at PLOS Biology. However, the reviewers do raise important points that will need to be thoroughly addressed in a revision. Primarily, Reviewer 2 has concerns about the analysis and power calculations, which will need to be fully and satisfactorily addressed. Both reviewers also point out that, given the broad potential interest in this article across fields, it needs to be made much more understandable. The editorial team and Academic Editor all agree that a major rewrite is needed for this to be suitable for our broad readership. Overall, while we will not be able to accept the current version of the manuscript, we would welcome re-submission of a revised version that takes into account the reviewers' comments.

*We are happy with the overall positive evaluation of our manuscript and addressed your comments and the reviewers’ main comments below. We also made some additional changes in the text to make our manuscript more understandable for a wider audience.*

Please note that we cannot make any decision about publication until we have seen the revised manuscript and your response to the reviewers' comments. Your revised manuscript is also likely to be sent for further evaluation by the reviewers.

We expect to receive your revised manuscript within 2 months.

Please email us ([plosbiology@plos.org](mailto:plosbiology@plos.org)) if you have any questions or concerns, or would like to request an extension. At this stage, your manuscript remains formally under active consideration at our journal; please notify us by email if you do not intend to submit a revision so that we may end consideration of the manuscript at PLOS Biology.

**IMPORTANT - SUBMITTING YOUR REVISION**

Your revisions should address the specific points made by each reviewer. Please submit the following files along with your revised manuscript:

1. A 'Response to Reviewers' file - this should detail your responses to the editorial requests, present a point-by-point response to all of the reviewers' comments, and indicate the changes made to the manuscript.

*NOTE: In your point by point response to the reviewers, please provide the full context of each review. Do not selectively quote paragraphs or sentences to reply to. The entire set of reviewer comments should be present in full and each specific point should be responded to individually, point by point.

You should also cite any additional relevant literature that has been published since the original submission and mention any additional citations in your response.

2. In addition to a clean copy of the manuscript, please also upload a 'track-changes' version of your manuscript that specifies the edits made. This should be uploaded as a "Related" file type.

*Re-submission Checklist*

When you are ready to resubmit your revised manuscript, please refer to this re-submission checklist: <https://plos.io/Biology_Checklist>

To submit a revised version of your manuscript, please go to <https://www.editorialmanager.com/pbiology/> and log in as an Author. Click the link labelled 'Submissions Needing Revision' where you will find your submission record.

Please make sure to read the following important policies and guidelines while preparing your revision:

*Published Peer Review*

Please note while forming your response, if your article is accepted, you may have the opportunity to make the peer review history publicly available. The record will include editor decision letters (with reviews) and your responses to reviewer comments. If eligible, we will contact you to opt in or out. Please see here for more details:

<https://blogs.plos.org/plos/2019/05/plos-journals-now-open-for-published-peer-review/>

*PLOS Data Policy*

Please note that as a condition of publication PLOS' data policy (<http://journals.plos.org/plosbiology/s/data-availability>) requires that you make available all data used to draw the conclusions arrived at in your manuscript. If you have not already done so, you must include any data used in your manuscript either in appropriate repositories, within the body of the manuscript, or as supporting information (N.B. this includes any numerical values that were used to generate graphs, histograms etc.). For an example see here: <http://www.plosbiology.org/article/info%3Adoi%2F10.1371%2Fjournal.pbio.1001908#s5>

*Blot and Gel Data Policy*

We require the original, uncropped and minimally adjusted images supporting all blot and gel results reported in an article's figures or Supporting Information files. We will require these files before a manuscript can be accepted so please prepare them now, if you have not already uploaded them. Please carefully read our guidelines for how to prepare and upload this data: <https://journals.plos.org/plosbiology/s/figures#loc-blot-and-gel-reporting-requirements>

*Protocols deposition*

To enhance the reproducibility of your results, we recommend that if applicable you deposit your laboratory protocols in protocols.io, where a protocol can be assigned its own identifier (DOI) such that it can be cited independently in the future. For instructions see: <https://journals.plos.org/plosbiology/s/submission-guidelines#loc-materials-and-methods>

Thank you again for your submission to our journal. We hope that our editorial process has been constructive thus far, and we welcome your feedback at any time. Please don't hesitate to contact us if you have any questions or comments.

Sincerely,

Hashi Wijayatilake, PhD,

Managing Editor

PLOS Biology

*****************************************************

REVIEWS:

**Reviewer #1 (Matthew Page):**

This is a really interesting study addressing an important question: does a more detailed template for registering study plans lead to a more transparent plan? The methods are rigorous and documented in sufficient detail to allow others to replicate the study. The authors did a great job responding to the reviewer comments at the other journal, but I have a few other comments that I hope will improve the manuscript.

General comment: The term preregistration is used throughout. I am from medicine where we have used the terms prospective registration or retrospective registration for decades, so I find the "pre" added to this term confusing. The use of "pre" implies some act done before registering a study, but I'm not sure what that act is. A clarification about terminology would be great, particularly for health and medical researchers who would also benefit from reading this work.

*Response: thank you for this valuable comment. We changed the order of the second and third paragraph in the introduction and added the following sentence to explain how preregistration and prospective registration are related: “Preregistration is, therefore, comparable to prospective registration of clinical trials (De Angelis et al., 2004; Nosek, Ebersole, DeHaven, & Mellor, 2018; Rice & Moher, 2019), although with an added focus on registering planned analyses.” (row 56-59)*

Abstract: The authors claim that "Opportunistic use of 'researcher degrees of freedom' aimed at obtaining statistical significance increases the likelihood of obtaining false positive results and overestimated effect sizes, and lowers the replicability of published results." I agree with the first part of the sentence, but not with the second (i.e. "…lowers the replicability of published results"). Incomplete reporting and not sharing study materials lowers the ability for others to replicate a study, not use of 'researcher degrees of freedom'. But perhaps I am interpreting "replicability" differently to the authors (given the definition varies so much!)

*Response: Thank you, for pointing out this ambiguous phrase. We meant that many published results can’t be replicated, because of the large number of false positives and inflated effect sizes. Because we already stated that opportunistic use of researcher degrees of freedom increases the likelihood of obtaining a false positive result, we changed the sentence to: “Opportunistic use of ‘researcher degrees of freedom’ aimed at obtaining statistical significance increases the likelihood of obtaining and publishing false positive results and overestimated effect sizes” (row 24-26)*

Abstract: The authors claim that "Results of comparing random samples of 53 preregistrations from each format indicate that neither format restricted all researcher degrees of freedom and the Prereg Challenge format performed better on restricting degrees of freedom than the "Standard" format." The latter half of the sentence (re the Prereg Challenge format performing better) is not very clear (as a reader am I to interpret that this format was better for ALL researcher degrees of freedom, and how much better?). It also appears inconsistent with how you interpret the results in the Discussion.

*Response: You are right. To improve the clarity, we changed the sentence too: “. Results of comparing random samples of 53 preregistrations from each format indicate that the structured format restricted the opportunistic use of researcher degrees of freedom better (Cliff’s Delta = 0.49) than the unstructured format, but neither eliminated all researcher degrees of freedom” (row 35-38)*

Abstract: The authors claim that "We also found a very low concordance rate among coders about the number of hypotheses (about 15%), suggesting that preregistration is difficult and an acquired skill…" Sorry but I don't follow the logic here. Low concordance among raters appraising preregistrations implies that it is difficult for readers to understand the content of preregistrations or that the coders needed more training, not that that the act of preregistration itself is difficult, in my view. Perhaps clarification on this point would be useful.

*Response: Thanks again. To clarify this point, we changed this sentence to: “We also observed very low concordance rate among coders about the number of hypotheses (14%), indicating that they are often not clearly stated“ (row 38-39). In the limitation section we extended our discussion on the different reasons of such a low concordance rate and also state that the coders might have needed more training:*

*“Also, some preregistrations were so ambiguous that it was hard to make sense of the planned research. For example, it was frequently difficult to understand what the main hypotheses were (e.g., multiple dependent variables are stated in one sentence, which might indicate one multivariate hypothesis, or multiple hypotheses that are tested with separate tests), as demonstrated by the percentage of agreement on the number of hypotheses in a preregistration being extremely low (around 14%). This might indicate that raters need more training.” (row 555-561)*

Introduction: The authors state that "Specification of the design and the analysis plan before observing the outcomes prevents the outcomes from affecting design and analysis decisions." However, no empirical evidence to support this claim is provided, despite there being a vast literature on reporting biases in medicine that has examined this issue directly that should be acknowledged. Also the paragraph starting, "The potential value of preregistration has been known for a long time…" is also rather limited in my view as it makes no mention of the vast literature on the potential value and observed limitations of registration of clinical trials in medicine. Why not build on that past literature to frame your arguments in the current study? For example, we have known for many years via meta-research studies in medicine that basic registration isn't enough to prevent researcher degrees of freedom, and that detailed protocols and statistical analysis plans are essential (for which a reporting guideline by Gamble in JAMA exists). I realise you may want to focus only on psychology, but I just think that reflecting on this literature could make the rationale for the study stronger.

FOLLOW-UP COMMENT: Oh, I see you refer to this evidence in the Discussion. Hmm, seems a bit odd not to allude to this evidence in the Introduction.

*Response: Thank you. Originally, we had the idea to keep the introduction short. We have now added some references (Dwan et al., 2008; Kaplan & Irvin 2015; Kirkham et al., 2010; van der Steen et al., 2018) to the sentence:*

*“Specification of the design and the analysis plan before observing the outcomes prevents the outcomes from affecting design and analysis decisions (Dwan et al.; 2008, Kaplan & Irvin 2015; Kirkham et al., 2010; van der Steen et al., 2018).” (row 59-61)*

*Furthermore, we changed the sentence about the potential value of preregistration to:*

*“The potential value of preregistration has been known for a long time (de Groot, 1956/2014) and has become common for clinical trials (De Angelis et al., 2004) with inconsistent emphasis on registration before the study and pre-specification of analysis plans (Zarin, Tse, Williams, & Rajakannan, 2017). The practice is now gaining popularity in other fields, particularly the social and behavioral sciences like psychology (Christensen et al., 2020; Nosek & Lindsay, 2018).” (row 102-107)*

Methods - Variables of interest: I wonder if an alternative to "Restriction Score" could be used. It has a pretty negative connotation I think, with a possible interpretation of this study being that "preregistration restricts researchers!". Why not something more positive like a "Transparency Score", thus leading to the more positive conclusion that "preregistration improves transparency!".

*Response: Thank you for your suggestion! We changed the ‘Restriction Score’ to ‘Transparency Score’. We added a note in which we state: “These were called ‘Restriction Scores’ in the preregistration as it concerns descriptions that restrict opportunities for researcher degrees of freedom, but as these descriptions entail transparency about the research process we use ‘Transparency Scores’. We thank a reviewer for this suggestion.” (note 2)*

Results: Minor quibble - there are too many abbreviations in the Results section (e.g. DF, PCR, SPR, DV, IV) which makes it hard to follow. A case in point is the following sentence, which I find dizzying to read: "Higher scores were obtained by PCR than by SPR on DFs that pertained to the operationalization of the variables in the study: D1 ('multiple manipulated IVs', D = 0.22), D3 and A5 ('multiple measures DV' and 'select DV measure', D = 0.19), and D5 and A12 ('additional IVs exclusion' and 'in/exclusion criteria', D = 0.24).". I see no problem with spelling everything out in full. Also, for a medical researcher like me, PCR means something else entirely!

*Response: We agree that we ended up with many abbreviations and have now spelled out the abbreviations RDF, DF, IV and DV. To improve readability and the understanding of our paper we also changed PCR and SPR to structured and unstructured format.*

Table 2. I'm sure this is reported elsewhere but from just reading the table it's unclear to me what the range of possible scores is in the Mean (SD) columns (e.g. is a mean of 1.98 large or small?). A footnote would help.

*Response: We added to the note of Table 2 that the mean scores per degree of freedom can range from 0 to 3.*

--

**Reviewer #2 (Ulf Toelch, QUEST Center for Transforming Biomedical Research):**

The study by Vanderkamp et al. addresses an important and timely subject namely how current practices in preregistration influence the depth and detail of preregistrations. I also appreciate the openness and transparency to publish the previous reviewer comments. The material on the OSF repository seems complete, I did however not review the code. The manuscript is not an easy read and could still need some editing towards readability. Particularly some procedures are only clear if you read the R code or the supplementary coding scheme. Beyond this I have some major concerns particularly about the analysis that I would like to see clarified.

1. This is reflecting on a point one of the previous reviewers as to the transition from preregistration to manuscript. A recent preprint at least hints at the possibility that adherence to protocols may not be optimal (10.31234/osf.io/d8wex). It is thus even more important to have high standards for preregistration. It will be harder to adhere to a preregistration with full analysis plan than to a preregistration stating only a hypothesis. It is, however, a lot more work for reviewers to connect a preregistration to a manuscript if the preregistration is long and exhaustive. I feel that the last part of the discussion puts little emphasis on the fact that a. writing good preregistrations is hard b. reviewing them puts a considerable additional burden on reviewers. A more balanced view on preregistration would be warranted and cases where preregistration may not be applicable could be stated.

*Response: Thank you for your valuable suggestions. Note that we also indicate in the last sentence of the abstract that “effective preregistration is challenging, and registration formats that provide effective guidance may improve the quality of research. “*

*We decided to further discuss the subjective experiences with preregistration somewhat further and referred to a recent survey of participants who recently preregistered a study (Toth et al., 2020). Specifically, we added the following to the discussion*

*“Recently, participants who had completed a preregistration were asked about their experiences with preregistration in a survey (Toth et al., 2020). According to the participants, “preregistration encourages a more thoughtful planning process which leads to better-executed studies” (p. 19; Toth et al.) and they expected that it will increase the confidence in the study by journal editors. On the other hand, participants of this survey also noted some possible disadvantages, like the amount of time it takes to write a preregistration and that they are unsure what to do with (reasonable) changes from the preregistered plan, which shows the need for more guidance of researchers who want to preregister a study. It is also not known yet, whether preregistrations puts a substantial extra burden on reviewers. It will be productive to investigate potential tradeoffs of overall research efficiency, quality, and progress in the context of preregistration and the extent of their specificity and exhaustiveness.” (row 600-610)*

*And:*

*“When there is no clear theory, or when the data or the analyses are quite complex, it could be more efficient to begin with exploratory analysis despite the costs to diagnosticity of statistical inferences.” (row 614-616)*

2. The list of DFs by Wicherts et al. is at least partly targeted specifically at psychology. As PLOS Biology has a broad readership it will be interesting from which fields the preregistrations stem. My guess is that preregistrations will be mainly from psychology. This could be another limitations as e.g. animal experiments may need a different focus for preregistration (<https://www.animalstudyregistry.org>)

*Response: We agree that the list of DFs might not be applicable for all research types and have added the following sentences to the discussion:*

*“Another limitation is that most preregistrations (88.6%) were from psychology. This partly reflects the fact that preregistrations popularity has grown most quickly in this field. The researcher degrees of freedom coding format was developed in the context of social-behavioral research providing good matching for the purposes of this research (Wicherts et al., 2016). To evaluate the generalizability of our findings to other fields, it will be worth reviewing and updating the coding format for unique methodological issues in other disciplines. New registration formats have emerged for other research activities such as animal studies (*[*https://www.animalstudyregistry.org*](https://www.animalstudyregistry.org)*;* [*https://www.preclinicaltrials.eu*](https://www.preclinicaltrials.eu)*), qualitative research (Haven & Van Grootel, 2019), neuroimaging (Flannery, 2018), and cognitive modeling (Cruwell & Evans, 2019). These offer productive opportunities for extending this research to new domains and preregistration formats (see https://osf.io/zab38/ for a curated list).” (row 538-548)*

3. I do not find the analysis very compelling.

A. The power analysis is done on a Cohens d of .5 You state that as you do not have any prior knowledge about the ES. One than must assume that you think that .5 will be a meaningful effect size. This corresponds to a Cliffs d of .33. This means that only effect sizes equal/larger should be considered as meaningful difference (not only statistically significance). How will smaller effect sizes make a difference between the two preregistration schemes? And if they do why did you not power for them? See my summary point below also.

*Response: We calculate and label effect sizes smaller than d = .5. Because we ‘powered’ our design for this medium effect size estimate, one cannot make strong inferences about smaller effect sizes (and we do not do so in our main text); as these effect sizes’ precision is relatively low, we generally cannot reliably distinguish them from 0.*

B. It is not clear whether power calculation was conducted on two or one sided test (perhaps I did not read this properly). Your main results is one sided (summary restriction scores) the single DFs are two sided. This needs clarification.

*Response: Thank you for your comment. To clarify, our only (directional) confirmatory hypothesis is about the difference in Restriction Score between the two registration types. Thus the power analysis was based on this analysis and is based on a one-sided test. As stated in our preregistration, we did exploratory follow-up analyses to check for differences between the two registrations types for each degree of freedom separately. Because this was exploratory, these tests were two-tailed. Because this might not be clear from the text, we added to the power analysis in our main text that this was a one-tailed test. In the statistical analysis we explain that the exploratory degree of freedom level analyses are based on two-tailed tests:*

*“We then conducted preregistered follow-up analyses to investigate on which degrees of freedom the two preregistration types differed. We conducted 29 preregistered two-tailed Wilcoxon-Mann-Whitney U-tests to compare the median scores of the two formats per degree of freedom.” (row 282-287)*

C. Your main result (summary restriction scores) is calculated on the mean of median scores. This is not reflected in the power analysis. Some DFs can only have values of 0, 2, and 3, some can have values of 0, 1, 2, 3. As this is your main result your choice to first calculate the median and then the mean needs more motivation. Perhaps you could conduct a non parametric bootstrap to confirm your result. It is also not clear to me what the calculated effect size here means. What would be a viable interpretation?

*Response: Our power analysis does correspond to our main analysis. The summary Restriction Scores (now called Transparency Scores) are not the mean of the median scores. As stated in the paragraph about the variables of interest, the Restriction Score is calculated as follows:*

*“This Transparency Score was computed as the unweighted arithmetic mean of the scores (0-3) of all 29 researcher degrees of freedom in our protocol.” (row 267-266)*

*This resulted in the distribution of the Restriction Scores as presented in the figure below. These Restriction Scores are compared between the two registrations types by means of a Wilcoxon-Mann-Whitney U-test, for which we did our power-analysis. As suggested, we performed a non-parametric bootstrap in which we compared the two medians. This resulted in a p-value of .001. Therefore, we added to the manuscript in the statistical analysis part:*

*“For exploratory analyses, we performed a non-parametric bootstrap procedure to compare the median Transparency Scores.” (row 291-292)*

*And in note 3 in the results section:*

*“An additional exploratory bootstrap procedure compared the medians of original Transparency Scores is consistent with the preregistered result (p = .001, two-tailed)”*

D. Table 2. Your data has a clear hierarchical structure (different categories) with a lot of depended data points (within study). This is not reflected in your simple multiple test two group comparison scheme. The preregistered analysis is simplistic and neglects a lot of these effects. What are correlations within study? Will a preregistration that is underreporting in one area also do this in another (as additional material)?

*Response: Thank you for your comment. Our preregistered main hypotheses was at the level of preregistered studies, and not on the level of degrees of freedom. Thus, the hierarchical structure of the data is not a problem at all for testing our main hypotheses. The results of our exploratory two-group comparisons at the level of degrees of freedom may indeed be statistically dependent. We examined this statistical dependency after calculating Restriction Scores for the different categories and computed correlations to investigate the associations between the different categories. We found significant correlations between Design, Data Collection, and Data Analysis (see the exploratory analyses section for more details).*

*“Furthermore, we calculated Transparency Scores for each sub-category (e.g., design, data collection) separately.” (row 274-275)*

*“Furthermore, the Transparency scores on the subsets of items created for exploratory purposes are compared with the two-tailed two-group Wilcoxon-Mann-Whitney U-test consistent with the other analyses. We explored the association between the Transparency scores of the different sub-categories with Spearman’s rank order correlations.” (row 292-294)*

*“Transparency Scores were calculated for each sub-category of the coding measure. Two-tailed Wilcoxon-Mann-Whitney U-tests showed significant large differences in Transparency Scores for the sub-category Design (Unstructured mdn = 0.57; Structured mdn = 0.86; U = 1930, p < .001; Cliff’s Delta = 0.40) and Data Analysis (Unstructured mdn = 0.82; Structured mdn = 0.49; U = 2148, p < .001; Cliff’s Delta = 0.56). The Design Transparency Score was correlated with the Data Collection Transparency Score (r_s_ = .478, p < .001) and with the Data Analysis Transparency Score (r_s_ = .794, p < .001). Data Collection Transparency Scores were correlated with Data Analysis Transparency Scores (r_s_ = .354, p < .001).” (row 457-464)*

Additionally you state: "We then conducted exploratory follow-up analyses to investigate on which DFs the two preregistration types differed." in Material and Methods

And: "As preregistered, we conducted follow-up analyses to examine on which of the DFs there were statistically significant differences in the distribution of scores between SPR and PCR." in Results

Does this mean that the whole Table 2 is exploratory and not confirmatory? But is it preregistered? This could be an error in my reading but as it is this is confusing and does not allow a critical appraisal of the results in Table 2. The results also showcase the pitfalls of prespecifying analyses where the data structure is complicated. If I prespecify a suboptimal analysis scheme in a preregistration and reviewers want significant changes will this then result in entirely exploratory analyses in the final paper?	I am unsure about the last point myself but want to mention it here to stimulate discussion.

*Response: We understand the confusion. Our main preregistered hypothesis was about the Restriction Score and, as preregistered, we did follow-up analyses on the degree of freedom level (results presented in Table 2). We made this clearer, by calling these follow-up analysis not exploratory (see row 359), and add to the subheadings “main analysis” and “follow-up analyses”, and only use exploratory analyses for the analyses that we preregistered as such and the analyses you and other reviewers suggested. The results of these exploratory analyses are also only presented in the section about exploratory analyses.*

*People might of course disagree about whether the prespecified analyses are the best possible analyses. Therefore, it is advisable to report other possible ways to analyze the data in the explorative analysis section as we did. This can be seen as a sensitivity analysis. When the authors themselves have good reasons to dismiss their prespecified analysis, we think it is still good to report the results of the prespecified analysis, if possible, for transparency. This can then be accompanied with the preferred analysis results. This will of course not count as preregistered results but can be still valuable as an exploratory result. Transparency is in all these situations key. We elaborate a bit more on this in the conclusion:*

*“Preregistration does not imply that confirmatory analyses are good and exploratory analyses are bad. The goal of preregistration is to be transparent and make clear which is which. When there is no clear theory, or when the data or the analyses are quite complex, it could be more efficient to begin with exploratory analysis despite the costs to diagnosticity of statistical inferences. Further, exploratory analysis is important and valuable for generating hypotheses from observed data. Indeed, many important discoveries emerged from exploratory analysis in an unanticipated pattern of results. This can lead to a new hypothesis or a new analysis method that is then tested in new data. Preregistration does not tie the researchers’ hands; it makes transparent the research process toward more trustworthy and replicable results. Having clear, specific, and comprehensive preregistrations and efficient workflows that support producing them may facilitate better understanding and rigor of exploratory and confirmatory research, and ultimately accelerate progress.” (row 612-623)*

To summarise, I would have wished for a result reporting and discussion based on the effect sizes. What does it mean if there is a difference between the two scores? What is the consequence of such a difference for the quality of the pre-registration. For ordinal variables an argumentation based on the mean values should be avoided. I find the percentages per ordinal level quite illustrative and a Figure for cases like randomisation and missing data, etc. could be helpful. In this context I miss the connection to survey data on QRPs. It would benefit the paper if you could relate the base rates of QRPs that you find for preregistrations to findings from the literature on self reported QRPs (Loewenstein and others). This would also address some of the concerns of previous reviewers and contextualise your results better. I know that space is limited but you could point out where preregistrations are particularly successful and where there is additional effort necessary.

*Response: thank you for your last comments. As stated in the part about the scoring protocol: “We assigned scores from 0 to 3 to each degree of freedom for each preregistration: opportunistic use of the degree of freedom was (0) not restricted at all; (1) restricted to some degree; (2) completely restricted (i.e., it was ‘precise’); (3) completely restricted and exhaustive (i.e., the preregistration included an explicit statement that no deviation from the way it was registered would occur).”*

*We made some changes in the result section to better interpret the values and differences that we have found (e.g., “Both formats completely restricted (a Transparency Score of 2) this degree of freedom (Unstructured: mean = 1.98; Structured: mean = 2.02).”; row 440-442)*

*Furthermore, we added a figure to the Appendix to summarize the results presented in Table 2.*

*And thank you for the suggestion to relate our results with common QRPs as described by John et al. We added the following to our results:*

*“Transparent reporting of these degrees of freedom can prevent Questionable Research Practices like failing to report all dependent variables, failing to report all of a study’s conditions, and informed exclusion of data (self-admittances rates of 63.4%, 27.7%, and 38.2%, respectively; John et al., 2012).” (row 380-383)*

I sign my reviews

Ulf Toelch, QUEST Center for Transforming Biomedical Research

__________________________________________________

In compliance with data protection regulations, you may request that we remove your personal registration details at any time. (Use the following URL: <https://www.editorialmanager.com/pbiology/login.asp?a=r>). Please contact the publication office if you have any questions.
